# Keratometry Changes Between Year Seven and Twelve After Corneal Crosslinking in Patients with Keratoconus

**DOI:** 10.3390/jcm14082585

**Published:** 2025-04-09

**Authors:** Lukas Neuhann, Diana Vogel, Jens Dreyhaupt, Adnan Kilani, Christian Enders

**Affiliations:** 1 Department of Ophthalmology, LMU University Hospital, LMU Munich, 80336 Munich, Germany; 2 MVZ Prof. Neuhann, 80637 Munich, Germany; 3 Institute of Epidemiology and Medical Biometry, Ulm University, 89075 Ulm, Germany; 4 Department of Ophthalmology, Ulm University, 89075 Ulm, Germany

**Keywords:** corneal crosslinking, keratoconus, keratectasia, keratoplasty

## Abstract

**Background/Objectives**: To evaluate the timing and extent to which late keratometric changes can occur between year 7 and 12 after corneal collagen crosslinking (CXL) in patients with keratoconus. **Methods**: A subgroup of a retrospective cohort study of all consecutive patients who underwent CXL at our cornea center between 2007 and 2011 was analyzed. The inclusion criteria consisted of CXL according to the Dresden protocol and a full set of keratometry parameters collected by Scheimpflug tomography preoperatively and at year 7, 9 and 12 after CXL. **Results**: A total of 46 eyes of 35 patients were included. The most relevant keratometric parameters (Kmax, TCT, K1, K2 and anterior astigmatism) decreased statistically significantly at year 7 after CXL, while there was no relevant difference for posterior astigmatism and the flat axes of anterior and posterior astigmatism. All keratometric parameters (except for K2) remained stable between year 7 and 12 without statistically significant change, according to mixed effect model regression analysis. BCVA improved statistically significant between the baseline and year 7 and remained stable until year 12. Suspected disease progression was noted in two patients (4.3%) between year 7, 9 and 12 post-CXL. **Conclusions**: Keratometric and functional results improve significantly 7 years after CXL in comparison to preoperative values and show very effective stabilization without clinically relevant changes up to year 12. However, while the risk of disease progression decreases remarkably after 7 years, in rare cases, suspected progression can occur even up to year 12. Therefore, regular control visits with keratometry measurements are advisable at least every 2 to 3 years in the late postoperative course.

## 1. Introduction

Keratoconus is a leading indication for penetrating keratoplasty (PKP) or deep anterior lamellar keratoplasty (DALK) [1,2]. Since the development of corneal crosslinking (CXL), keratoconus progression can be halted at an early stage, reducing the risk of visual deterioration and the need for keratoplasty [3,4,5]. The effectiveness and safety of CXL have been well-documented in short- to mid-term studies [6,7,8,9,10] and long-term studies, with follow-ups extending up to 15 years [11,12,13]. Modifications to the original “Dresden protocol”, including accelerated procedures to shorten the patient’s treatment time [14,15], transepithelial procedures [16,17], pulsed CXL for better oxygen diffusion into the stroma [18], customized CXL [19] and adapted fluence to treat thinner corneas [20] continue to be explored to optimize outcomes.

While the indication to perform CXL is now routine in clinical practice, no sufficient answer can yet be given regarding the necessity of further controls in the long-term course. Since patients are usually relatively young when crosslinking is performed, it is necessary to know in which time interval corneal changes, which can lead to functional changes, can still be expected years after crosslinking. We have already reported detailed data about the course and stability between the first and seventh year after CXL in a large group of patients, showing that even after 7 years, changes in keratometric and functional parameters still take place [21]. Subsequently we, therefore, performed a retrospective analysis of a smaller cohort of patients that were followed up even longer, with keratometric measurements 7, 9 and 12 years after crosslinking at the same cornea center.

## 2. Materials and Methods

We performed a retrospective monocentric cohort study. Therefore, the sample size was chosen due to feasibility. We included all patients in the analysis who received crosslinking at a single corneal center between 1 January 2007 and 30 December 2011 and met the other inclusion criteria. The study protocol was reviewed by the responsible ethics committee of the Bavarian State Medical Association. It decided that ethics approval was not required for this study and waived the need for informed consent, according to national medical regulations on retrospective monocentric clinical trials. All principles of the Declaration of Helsinki were followed.

The inclusion criteria included the performance of the CXL according to the Dresden protocol, the presence of a complete keratometry data set (KMax, K1, K2, anterior astigmatism, posterior astigmatism, thinnest corneal thickness (TCT)) collected by a Scheimpflug camera (Pentacam HR; Oculus Optikgeräte GmbH, Wetzlar, Germany) both preoperatively (baseline, BL) and 7 (Y7), 9 (Y9) and 12 years (Y12) after CXL, resulting in a minimum follow-up time of 12 years. Incomplete keratometry records, patients with history of previous corneal surgery and indications for CXL other than keratoconus were excluded.

Furthermore, we analyzed the development of the best-corrected visual acuity (BCVA), if available, which was obtained at BL, Y7, Y9 and Y12 with refractive determination using subjective refractometry. The collected values were then converted to logMAR.

The CXL was performed according to the Dresden protocol [4] and has been outlined in a previous evaluation of center data [11]. Parts of this patient population have already been included in comprehensive earlier evaluations [11,21].

The indication for CXL was made in clinical practice by an experienced ophthalmologist and cornea specialist.

Continuous data were expressed as mean, standard deviation (SD), minimum and maximum. Ordinal and categorical data were analyzed as absolute frequencies and percentages. Comparisons between different time points were performed using the t-test for dependent variables and mixed effects model regression analysis. A two-sided *p* value ≤ 0.05 was considered statistically significant. Due to the exploratory nature of this study, all results from statistical tests must be interpreted as hypothesis generating rather than confirmatory. Statistical analysis was performed using Microsoft Excel 2021 (Microsoft Corp, Redmond, WA, USA) and SAS, version 9.4 (SAS Institute, Cary, NC, USA).

## 3. Results

### 3.1. Demography

A total of 46 eyes of 35 patients with complete keratometry data sets who had received CXL and met the inclusion criteria were identified. The mean age of the patients at the time of CXL was 25.46 years with a standard deviation of 7.84 years. The oldest patient was 46.41 years old, and the youngest patient was 14.00 years old. Of these patients, 26 were male (74%) and 9 were female (26%). An overview of the keratometry values from BL, Y7, Y9 and Y12 is presented in Table 1.

### 3.2. BCVA Development

At BL, the mean BCVA of this subgroup was 0.26 logMAR with a standard deviation of 0.19 logMAR. At Y7, BCVA improved to 0.14 logMAR (SD: 0.17 logMAR). The improvement of mean BCVA at Y7 compared to the baseline is statistically significant (*p* < 0.01).

Mean BCVA improved to 0.12 logMAR (SD: 0.16 logMAR) at Y9 and to 0.13 logMAR (SD: 0.16 logMAR) at Y12. Mixed effects model regression analysis showed that the change in BCVA between Y7, Y9 and Y12 was statistically not significant.

### 3.3. Keratometry Development

Table 1 shows the development of keratometric values at BL, Y7, Y9 and Y12. As illustrated, Kmax, TCT, K1, K2 and anterior astigmatism decreased statistically significantly at Y7 as compared to BL, while there was no relevant difference in posterior astigmatism and the flat axes of anterior and posterior astigmatism.

**Figure 1:**
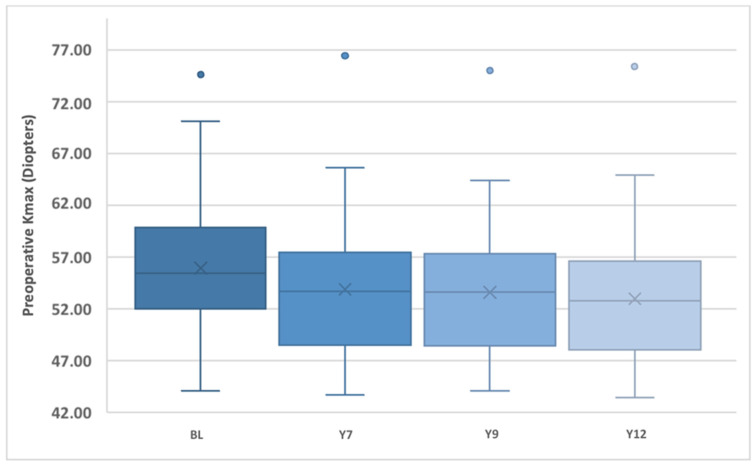
Course of Kmax at BL, Y7, Y9, Y12. Mean Kmax (represented by X) and the spread of minimum and maximum values are displayed. BL: baseline; Y7: year 7 postoperative, Y9: year 9 postoperative; Y12: year 12 postoperative.

Mean Kmax at Y7 was 53.87 dpt (SD: 6.23 dpt, maximum: 76.50 dpt, minimum: 43.70 dpt). At Y9, mean Kmax improved to 53.58 dpt (SD: 6.00 dpt, maximum: 75.00 dpt, Minimum: 44.10 dpt) and at Y12, mean Kmax improved to 52.97 dpt (SD: 6.24 dpt, maximum: 75.40 dpt, minimum: 43.40 dpt). Mixed effects model regression analysis showed that the change in Kmax between Y7, Y9 and Y12 was statistically not significant. Figure 1 shows the development of Kmax for all analyzed time points.

For all the remaining relevant keratometric parameters, mixed effects model regression analysis also revealed no statistically significant change between Y7, Y9 and Y12—except for K2, which decreased statistically significantly between Y7–Y12 (*p* = 0.04).

### 3.4. Progression and Adverse Events

In this patient cohort, two eyes (4.3%) exhibited suspected disease progression in terms of an increase in Kmax of ≥1 dpt between Y7 and Y12. Kmax at the baseline of these eyes was 52.90 dpt and 56.20 dpt compared to 55.97 dpt (SD: 6.16 dpt, maximum: 74.60 dpt, minimum: 44.10 dpt) in non-progressive eyes. The threshold an increase of ≥1 dpt in Kmax was chosen in accordance with previously reported long-term results after CXL [13,22,23] for best possible comparability.

The age of the two patients that showed an increase in Kmax ≥ 1 dpt was 21.36 and 38.59 years, whereas in non-progressive eyes it was in the mean 25.34 (SD: 7.74 dpt, maximum 46.41, minimum 14.00).

**Figure 2:**
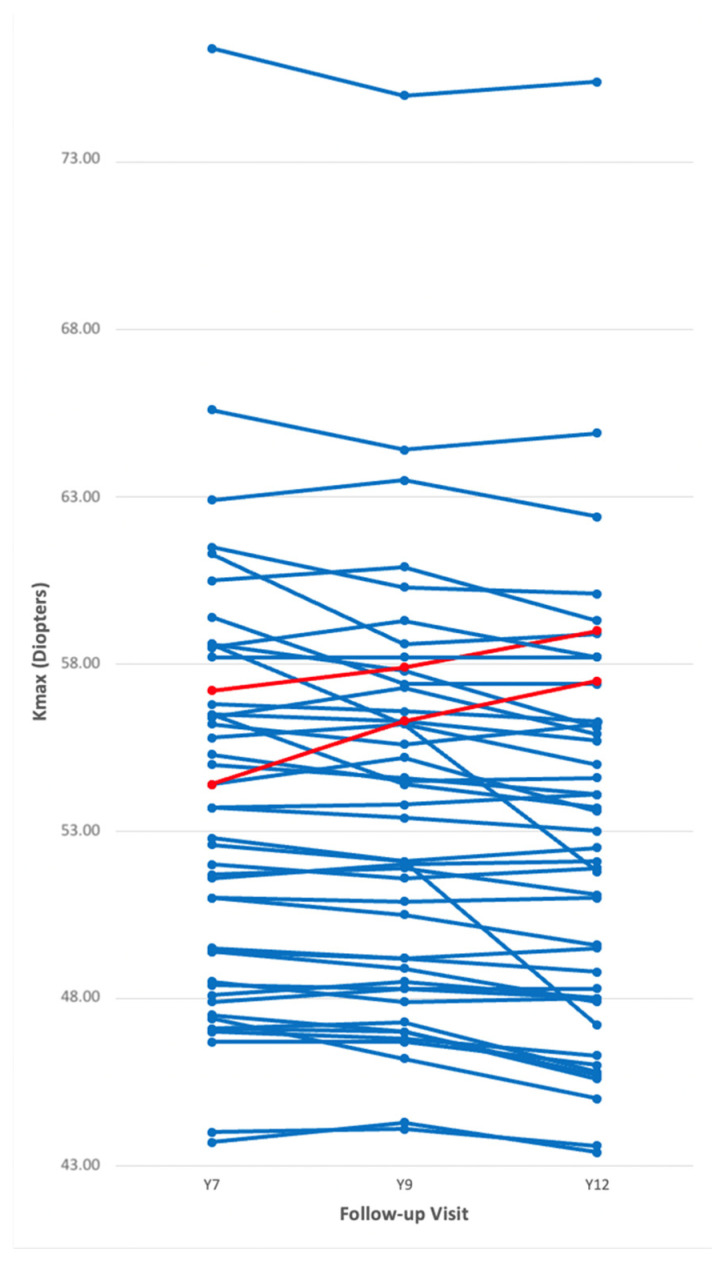
Each line represents a specific patient of the study cohort. It shows development of Kmax during each follow-up period and distribution of non-progressive cases (blue lines) and progressive cases (red lines).

The detailed course of each patient is illustrated in Figure 2. In one case, the increase in Kmax occurred between Y7 and Y9 (1.90 dpt) and again between Y9 and Y12 (1.20 dpt). In the other case, the increase in Kmax could be measured between Y9 and Y12 (1.10 dpt). In both cases, clinical decision was made against repeat CXL and patients are monitored closely.

In nine cases (19.6%), anterior astigmatism changed by 1 dpt or more, with three cases showing an increase and sic cases showing a decrease of ≥1 dpt. In three patients, this change in astigmatism occurred between Y7–Y12, in two patients between Y7–Y9 and in four patients between Y9–Y12. In 13 eyes (28.3%), the flat axis of anterior astigmatism changed by more than 10 degrees between Y7 and Y12. BCVA decreased by ≥0.1 logMAR in nine cases (19.5%) between Y7 and Y12.

## 4. Discussion

This study provides detailed insight into keratometric changes between years 7 and 12 post-CXLIn this retrospective analysis, we present detailed data about the long-term course and changes in keratometric measurements between the seventh, ninth and twelfth year after CXL.

The demographics of our cohort were within the known range regarding age and gender distribution and the baseline values of visual acuity and keratometry were comparable to already available studies [6,7,12,24]. The follow-up time of 12 years was relatively long compared to previous studies [5,6,7,8,22], but now studies of up to 15 years follow-up are available [13].

However, most studies with particularly long observation periods mainly compare results between the baseline or other single time points and final follow up, but do not analyze in detail to what extent and when exactly changes in keratometry take place during the observation period for a defined cohort; others are based on incomplete data sets, so that no true cohorts are available, or do not go into more detail about the composition of the data [8,12,22,25].

Our previous study showed that after initial improvement keratometry and functional parameters are generally stable for the first 5 years post-CXL, but even after 7 years further improvement is possible, while suspected progression mostly occurs in the first 3 years after treatment [21]. Since there was less recorded follow-up data over an even longer time period, we analyzed the extent to which keratometry changes took place between year 7, 9 and 12 after CXL for a separate subgroup of 46 eyes in order to gain further understanding about disease stabilization and refractive stability in the long term.

Mean BCVA of our patients showed marked improvement between BL and Y7 but showed no further statistically significant difference between Y7, Y9 and Y12, so that mean BCVA can be regarded as completely stable over the whole observation period between Y7 and Y12. This long-term stability in BCVA is coherent with previous studies [8,13], although one study also demonstrated regression to preoperative BCVA at the 9-year follow-up after initial postoperative improvement [25]. In our patient cohort, nine patients showed a decrease in BCVA of ≥0.1 logMAR between Y7 and Y12. The highest decrease in a single patient was 0.4 logMAR levels between Y7 and Y12; however, keratometry measurements were stable in this patient and the deterioration in BCVA was attributed to an exacerbation of dry eye disease. In all other cases, the decrease was 0.1–0.2 logMAR. Keratometry measurements were stable in all patients and no other ocular pathology could be diagnosed. Moreover, subjectively visual acuity was stable in all cases, so that according to our clinical experience we believe these small differences in BCVA measurements may—at least partly—be explained by fluctuations in subjective refractometry accuracy. Other potential causes include dry eye disease or lenticular changes; however, none of these were detailed in the medical records of the concerned patients.

Regarding the development of keratometry measurements, all relevant values except for posterior astigmatism showed statistically significant improvement after 7 years as compared to the baseline. After this, except for the decrease K2 between Y7 and Y12, there were no more statistically significant changes in any keratometry measurements between Y7, Y9 and Y12.

This confirms the excellent efficacy of CXL with long term stability and improvement after 7, 9 and 12 years postoperatively, which has already been published in the literature. Our results add a novel and more detailed aspect to the already existing data regarding long-term disease stabilization after CXL, since precise keratometric development and differences between different time points in the late postoperative course—for a whole cohort as well as each individual patient (Figure 2)—have not yet been explored, especially by applying a mixed models regression analysis for repeated measures.

In Nicula’s retrospective study, keratometry values were presented annually, which showed a steady decrease in Kmax after year 3 up to and including the 10th postoperative year [24]. However, while results at each postoperative year were compared to the baseline at year 3, in contrast to our results differences between single time points after year 3 were not analyzed. Other groups compared keratometry values after 1, 3, 5, 7, and 10 years, which confirmed the long-term efficacy of CXL but showed a large degree of fluctuation between Kmax measurements without a clear trend [8] and between year 1 and year 9 after CXL without any significant changes in Kmax development [25].

Other long-term results up to 10 years [22] and even 13 years [11,12] could also show steady and significant improvement of mean Kmax over the longtime periods; however, in these cohorts, comparisons were made only between the baseline and each postoperative year or only the last follow-up, but not for specific changes between every single follow-up visit after CXL. Recently, the first published 15-year results demonstrated that keratometry, corneal thickness and visual acuity remained stable between year 10 and year 15 post-CXL; the authors saw this as indication that corneal remodeling processes, such as corneal flattening, might not go on indefinitely [13]. A similar conclusion can also be drawn from our results: The changes in keratometry measurements between Y7, Y9 and Y12 were statistically not significant, except for K2—which was at the threshold of statistical significance with a *p*-value of 0.044—and also clinically irrelevant, since absolute values of Kmax, K1 and K2 decreased by less than 1 dpt, astigmatism only by 0.22 dpt and TCT by under 5 µm. Therefore, our results also indicate clinical stabilization in the late postoperative course rather than a significant ongoing remodeling process. Our analysis included patients between 14 and 46 years of age. Previous studies suggest that the response to CXL can vary significantly in pediatric and older patients [26]. In our cohort, there were four patients younger than 18 years and three patients older than 40 years. Because of the small sample size, a meaningful statistical subgroup analysis was not possible. However, the response to CXL in each of these seven patients was very close to mean development regarding keratometry values and BCVA of the whole cohort without any noticeable outliers. Also, in the two cases with suspected disease progression post-CXL age was 21 years in one patient and 38 years in the other. Therefore, in our cohort individual patient age had no obviously relevant influence on the outcome after CXL.

In accordance with the stabilization in mean Kmax, suspected disease progression, defined as an increase in Kmax of equal to or over 1 diopter, was only noted in two patients (4.3%) between Y7 and Y12. This rate is very low compared to other mid- to long-term studies [13,23,25] and also compared to our previously published data, where an increase in Kmax was seen in 22.2% between Y1 and Y7 post-CXL, with four out of five cases of progression being recorded between Y1 and Y3 [21]. Therefore, while most cases of suspected disease progression seem to take place in the first 3 years after CXL, our results indicate that the individual risk of progression decreases drastically in the late postoperative course. This is also in accordance with the results of Raiskup et al., who suggest that a lower failure rate between year 10 and year 15 post-CXL might be due to a natural tissue aging effect [13]. There was no clinically or statistically significant difference in the baseline Kmax or age between the two patients with Kmax increase > 1 dpt and the non-progressive patients. It is not clear why progression after CXL can occur even after 7 years of disease stabilization. Potentially eye rubbing could play an important role, since it is known to be a major risk factor for the progression of keratoconus [27]. However, this was not specifically documented in the medical records of the two patients with suspected progression.

Functionally, visual and refractive stability are very important for patients after CXL. No significant change was observed in the flat anterior and posterior axes. The maximum absolute change of anterior astigmatism was 0.22 dpt and for posterior astigmatism was 0.09 dpt. These changes were neither statistically significant nor clinically relevant, so that refractive outcomes can be regarded as stable between Y7 and Y12. This confirms previously published data also showing no significant changes in astigmatism for follow-up periods of up to 10 years [7,24,25]. Anterior astigmatism changed by ≥1 diopter in 19.6% of patients between Y7–Y12, with no trend for a specific time period. In 28.3%, the flat axis of anterior astigmatism changed by more than 10 degrees between Y7–Y12, which can be clinically relevant for patients.

Our study has limitations, which result from the retrospective study design without a control group and from a and possible selection bias because only patients with complete keratometric data sets at all time points were included. Future multicenter studies would be helpful to validate our results. However, the complete data set enabled us to perform a mixed effects model regression analysis instead of only pairwise comparison between single time points, which together with the comparably large sample size is a notable strength of our analysis.

To summarize, we can confirm the excellent long-term stability and safety of CXL in our cohort. Keratometric and functional results improve significantly after 7 years in comparison to preoperative values and show very effective stabilization up to year 12, maintaining improvement without clinically relevant changes in the absolute majority of cases. While the risk of disease progression generally seems to be remarkably lower after 7 years than in the first 3 postoperative years, in two patients suspected progression was still noted between Y7 and Y12. Therefore, it is advisable to perform regular control visits with keratometry measurements at least every 2 to 3 years even in the late postoperative course 7 to 12 years after CXL.

## Figures and Tables

**Table 1:** **Keratometry at BL, Y7, Y9 and Y12 (*n* = 46)**. BL: baseline; Y7: year 7 postoperative, Y9: year 9 postoperative; Y12: year 12 postoperative; SD: standard deviation; Max: maximum; Min: minimum; D: diopters; μm: micrometers; Kmax: maximum keratometry in the steepest meridian; TCT: thinnest corneal thickness, K1: keratometry in the flattest meridian; K2: keratometry in the steepest meridian; °: degrees; *p*: *p*-value; ^1^ comparison between baseline and Y7; ^2^ comparison between Y7 and Y9; ^3^ comparison between Y7 and Y12; ^4^ comparison between Y9 and Y12; **bold:** statistically significant.

		BL	Y7	Y9	Y12	*p* ^1^	*p* ^2^	*p* ^3^	*p* ^4^
Kmax (D)	Mean (SD)	55.91 (6.04)	53.87 (6.23)	53.58 (6.00)	52.97 (6.24)	**<0.001**	0.602	0.234	0.187
	Min/Max	44.10/74.60	43.70/76.40	44.10/75.00	43.40/75.40				
TCT (µm)	Mean (SD)	456.48 (50.00)	422.30 (57.78)	423.89 (57.50)	417.78 (59.31)	**<0.001**	0.810	0.493	0.354
	Min/Max	374.00/603.00	274.00/541.00	279.00/547.00	299.00/547.00				
K1 (D)	Mean (SD)	46.33 (3.92)	45.36 (4.50)	45.21 (4.53)	44.72 (4.67)	**0.012**	0.728	0.145	0.266
	Min/Max	38.90/56.00	37.50/54.80	36.90/55.00	36.00/54.40				
K2 (D)	Mean (SD)	49.77 (3.99)	48.22 (4.09)	48.02 (4.14)	47.36 (4.26)	**<0.001**	0.640	**0.044**	0.120
	Min/Max	41.80/57.70	39.90/56.40	39.80/56.30	39.40/55.80				
Anterior Astigmatism (D)	Mean (SD)	3.45 (1.89)	2.85 (1.65)	2.80 (1.57)	2.63 (1.61)	**0.018**	0.839	0.342	0.455
	Min/Max	0.60/9.00	0.60/8.30	0.60/8.60	0.10/8.10				
Flat Axis Anterior Astigmatism (°)	Mean (SD)	82.95 (57.31)	77.73 (62.22)	88.89 (72.25)	82.54 (61.71)	0.474	0.350	0.687	0.594
	Min/Max	0.90/173.30	0.80/178.30	0.10/345.50	0.80/179.40				
Posterior Astigmatism (D)	Mean (SD)	0.87 (0.45)	0.75 (0.43)	0.70 (0.38)	0.66 (0.40)	0.147	0.427	0.148	0.511
	Min/Max	0.10/2.30	0.10/1.80	0.00/1.60	0.00/1.60				
Flat Axis Posterior Astigmatism (°)	Mean (SD)	84.83 (58.33)	77.52 (66.74)	92.07 (65.47)	82.52 (66.00)	0.327	0.193	0.654	0.391
	Min/Max	5.10/176.00	0.30/178.20	1.40/179.90	0.10/175.30				

## Data Availability

The raw data supporting the conclusions of this article will be made available by the authors on request.

## References

[1] Hashemi H., Heydarian S., Hooshmand E., Saatchi M., Yekta A., Aghamirsalim M., Valadkhan M., Mortazavi M., Hashemi A., Khabazkhoob M. (2020). The Prevalence and Risk Factors for Keratoconus: A Systematic Review and Meta-Analysis. Cornea.

[2] Beltaief O., Farah H., Kamoun R., Ben Said A., Ouertani A. (2003). Penetrating keratoplasty in children. Tunis. Med..

[3] Spoerl E., Huhle M., Seiler T. (1998). Induction of cross-links in corneal tissue. Exp. Eye Res..

[4] Wollensak G., Spoerl E., Seiler T. (2003). Riboflavin/ultraviolet-a-induced collagen crosslinking for the treatment of keratoconus. Am. J. Ophthalmol..

[5] Raiskup-Wolf F., Hoyer A., Spoerl E., Pillunat L.E. (2008). Collagen crosslinking with riboflavin and ultraviolet-A light in keratoconus: Long-term results. J. Cataract Refract. Surg..

[6] Wittig-Silva C., Chan E., Islam F.M., Wu T., Whiting M., Snibson G.R. (2014). A randomized, controlled trial of corneal collagen cross-linking in progressive keratoconus: Three-year results. Ophthalmology.

[7] Hashemi H., Seyedian M.A., Miraftab M., Fotouhi A., Asgari S. (2013). Corneal collagen cross-linking with riboflavin and ultraviolet A irradiation for keratoconus: Long-term results. Ophthalmology.

[8] Seifert F.K., Theuersbacher J., Schwabe D., Lamm O., Hillenkamp J., Kampik D. (2022). Long-term outcome of corneal collagen crosslinking with riboflavin and UV-A irradiation for keratoconus. Curr. Eye Res..

[9] O’Brart D.P., Patel P., Lascaratos G., Wagh V.K., Tam C., Lee J., O’Brart N.A. (2015). Corneal cross-linking to halt the progression of keratoconus and corneal ectasia: Seven-year follow-up. Am. J. Ophthalmol..

[10] Taşçı Y.Y., Taşlıpınar Uzel A.G., Eyidoğan D., Saraç Ö., Çağıl N. (2020). Five-Year Long-Term Results of Standard Collagen Cross-Linking Therapy in Patients with Keratoconus. Turk. J. Ophthalmol..

[11] Enders C., Vogel D., Dreyhaupt J., Wolf W., Garip-Kuebler A., Hall J., Neuhann L., Werner J.U. (2023). Corneal cross-linking in patients with keratoconus: Up to 13 years of follow-up. Graefe’s Arch. Clin. Exp. Ophthalmol..

[12] Vinciguerra R., Pagano L., Borgia A., Montericcio A., Legrottaglie E.F., Piscopo R., Rosetta P., Vinciguerra P. (2020). Corneal cross-linking for progressive keratoconus: Up to 13 years of Follow-up. J. Refract. Surg..

[13] Raiskup F., Herber R., Lenk J., Ramm L., Wittig D., Pillunat L.E., Spoerl E. (2024). Corneal Crosslinking With Riboflavin and UVA Light in Progressive Keratoconus: Fifteen-Year Results. Am. J. Ophthalmol..

[14] Elbaz U., Shen C., Lichtinger A., Zauberman N.A., Goldich Y., Ziai S., Rootman D.S. (2015). Accelerated versus standard corneal collagen crosslinking combined with same day phototherapeutic keratectomy and single intrastromal ring segment implantation for keratoconus. Br. J. Ophthalmol..

[15] Lang P.Z., Hafezi N.L., Khandelwal S.S., Torres-Netto E.A., Hafezi F., Randleman J.B. (2019). Comparative Functional Outcomes After Corneal Crosslinking Using Standard, Accelerated, and Accelerated With Higher Total Fluence Protocols. Cornea.

[16] Epstein R.J., Belin M.W., Gravemann D., Littner R., Rubinfeld R.S. (2023). EpiSmart Crosslinking for Keratoconus: A Phase 2 Study. Cornea.

[17] Stulting R.D., Trattler W.B., Woolfson J.M., Rubinfeld R.S. (2018). Corneal crosslinking without epithelial removal. J. Cataract Refract. Surg..

[18] Mazzotta C., Traversi C., Caragiuli S., Rechichi M. (2014). Pulsed vs continuous light accelerated corneal collagen crosslinking: In vivo qualitative investigation by confocal microscopy and corneal OCT. Eye.

[19] Seiler T.G., Fischinger I., Koller T., Zapp D., Frueh B.E., Seiler T. (2016). Customized Corneal Cross-linking: One-Year Results. Am. J. Ophthalmol..

[20] Hafezi F., Kling S., Gilardoni F., Hafezi N., Hillen M., Abrishamchi R., Gomes J.A.P., Mazzotta C., Randleman J.B., Torres-Netto E.A. (2021). Individualized Corneal Cross-linking With Riboflavin and UV-A in Ultrathin Corneas: The Sub400 Protocol. Am. J. Ophthalmol..

[21] Neuhann L., Vogel D., Hall J., Dreyhaupt J., Werner J.U., Garip-Kuebler A., Enders C. (2024). Keratometry changes between year one to seven after corneal cross-linking in patients with keratoconus. Cornea.

[22] Raiskup F., Theuring A., Pillunat L.E., Spoerl E. (2015). Corneal collagen crosslinking with riboflavin and ultraviolet-A light in progressive keratoconus: Ten-year results. J. Cataract Refract. Surg..

[23] Mazzotta C., Traversi C., Baiocchi S., Bagaglia S., Caporossi O., Villano A., Caporossi A. (2018). Corneal collagen cross-linking with riboflavin and ultraviolet A light for pediatric keratoconus: Ten-year results. Cornea.

[24] Nicula C., Pop R., Rednik A., Nicula D. (2019). 10-Year results of standard cross-linking in patients with progressive keratoconus in romania. J. Ophthalmol..

[25] Derakhshan A., Heravian J., Abdolahian M., Bamdad S. (2021). Long-term outcomes of collagen crosslinking for early keratoconus. J. Ophthalmic Vis. Res..

[26] Vinciguerra R., Romano M.R., Camesasca F.I., Azzolini C., Trazza S., Morenghi E., Vinciguerra P. (2013). Corneal cross-linking as a treatment for keratoconus: Four-year morphologic and clinical outcomes with respect to patient age. Ophthalmology.

[27] Jaskiewicz K., Maleszka-Kurpiel M., Michalski A., Ploski R., Rydzanicz M., Gajecka M. (2023). Non-allergic eye rubbing is a major behavioral risk factor for keratoconus. PLoS ONE.

